# Epigenetic Mechanisms in Childhood Obesity

**DOI:** 10.1111/nyas.70045

**Published:** 2025-10-01

**Authors:** Guadalupe León‐Reyes, Génesis K. González‐Quijano, Francisco J. López‐Alavez, Maria Elizabeth Tejero

**Affiliations:** ^1^ Laboratory of Nutrigenetics and Nutrigenomics National Institute of Genomic Medicine (INMEGEN) Mexico City México; ^2^ SECIHTI‐INMEGEN Secretaria de Ciencia, Humanidades, Tecnología e Innovación Mexico City México

**Keywords:** childhood obesity, DNA methylation, endocrine disruptors, epigenetic mechanisms, microRNA

## Abstract

Epigenetic mechanisms, including DNA methylation, histone modification, and activity of noncoding RNAs (nc RNAs), affect the regulation of gene expression. These mechanisms are regulated by numerous environmental factors and are critical at specific windows of biological development, when organs and systems plasticity is increased. Evidence suggests that exposure to factors influencing these mechanisms may have an effect on health, including the risk for obesity at early stages of life. This study analyzed published evidence on the association between epigenetic mechanisms and childhood obesity. We searched for studies using untargeted detection methods followed by validation of associations between epigenetic mechanisms and obesity in children. Fifteen studies were found: two meta‐analyses on DNA methylation, seven original studies on DNA methylation, one systematic review on microRNAs, and five studies on nc RNA. No studies on histone modifications were identified. Most studies were conducted in blood cells or blood‐derived fluids. DNA methylation in different tissues was associated with childhood and adolescent obesity or related phenotypes, although comparison across studies is difficult due to technical differences. Nc RNA differed between children with and without obesity. Research on the role of factors regulating epigenetic mechanisms associated with childhood obesity is highly needed.

## 1 Introduction

Obesity is defined as abnormal or excessive fat accumulation that may impair health (World Health Organization) [1] and is a risk factor for the development of numerous chronic diseases. Obesity affects individuals of all age groups. Childhood obesity is associated with metabolic syndrome and type 2 diabetes in adolescence, a predictor for adult obesity and a risk factor for chronic diseases [2]. In children under 5 years of age, obesity is defined as weight‐for‐height greater than 3 standard deviations above the WHO Child Growth Standards median. In children between 5 and 19 years, obesity is defined as body mass index (BMI) for age greater than 2 standard deviations above the WHO Growth Reference median [1].

Obesity is a multifactorial disease influenced by genetic and environmental factors [3, 4, 5]. Numerous studies show that a group of genetic variants have been consistently associated with variation of BMI and common obesity in children and adolescents. However, these associations explain only a small part of the variation of these phenotypes and are not related to the increase in the prevalence of this disease [3]. Other factors, such as epigenetic mechanisms, have been proposed as possible contributors; a recent systematic review [5] identified over 140 different studies on the relationship between epigenetic mechanisms and childhood obesity, showing the increasing interest in the role of these mechanisms in human disease.

Epigenetic mechanisms produce mitotically heritable changes in gene expression that cannot be explained by modifications in the DNA sequence [6]. Gene expression regulation occurs in a tissue‐specific manner. The epigenetic mechanisms are: (1) DNA methylation; (2) histone modifications, which are considered as classic mechanisms that regulate transcription; and (3) noncoding RNAs (nc RNAs), which have some differences with the previously mentioned mechanisms because they regulate gene expression at the post‐transcriptional level, but do not produce heritable marks, also known as epigenetic marks [7, 8].

DNA methylation is the enzymatic incorporation of a methyl group on the 5´carbon of cytosines followed by guanine (cytosine‐phosphateguanine [CpG]), located at gene promoters and other regions of the genome. This process is regulated by DNA methyltransferases and ten‐eleven translocation methylcytosine dioxygenases that add and remove the methyl groups, respectively. Histone modifications are a group of enzymatically regulated reactions that add or remove functional groups (methyl, acetyl, and ubiquitin, among others) on specific amino acids located in histones, proteins bound to DNA, modifying the chromatin and regulating gene expression. These modifications are regulated by a group of enzymes, including histone acetyltransferases, histone deacetylases, and histone methyltransferases and demethylases [9]. Histone modifications may occur concomitantly with DNA methylation [10]. These chemical groups become marks that regulate the activity of groups of proteins that control gene expression. In some cases, these marks may be reversible and heritable.

Post‐transcriptional epigenetic regulation includes nc RNAs. This is a large family of molecules with widely diverse biological functions. According to their length in nucleotides (nt), there are small (<200 nt) and long noncoding RNAs (lncRNAs; >200 nt) [11]. MicroRNAs (miRNAs) belong to the small RNAs group, and their activity has been studied in the context of obesity [12]. miRNAs have been extensively studied because of their critical role in cell differentiation, apoptosis, metabolism, and development. miRNAs exert their regulatory effects by binding to the 3′ untranslated region of target miRNAs, resulting in translational repression or transcript degradation. They can be found in body tissues or fluids, like saliva, plasma, serum, and whole blood, encapsulated into extracellular vesicles or as circulating miRNA [13]. Many studies have explored the potential role of miRNAs as biomarkers of early childhood obesity, that is, profound deregulations in the circulating miRNA profile [14]. Obesity is also known to obstruct some miRNA‐mediated protective mechanisms that could modulate adipogenesis, adipose tissue browning, and autophagy inhibition [15]. Regarding nc RNA, its role in obesity is under research [16]. The interplay among the different epigenetic mechanisms remains to be investigated.

Numerous environmental factors may regulate epigenetic mechanisms associated with childhood obesity, including maternal health and nutritional status, diet, and exposure to drugs and pollutants [8, 17]. The role of epigenetic mechanisms in early life obesity has been investigated using a variety of study designs, mainly animal models. Exposures may occur before pregnancy, during the intrauterine phase and first years of life, when cells are going through differentiation and specialization processes and are more susceptible to changes influenced by environmental factors due to developmental plasticity [4, 5, 6, 7, 8, 9, 10, 11, 12, 13, 14, 15, 16, 17, 18]. Animal studies have shown permanent metabolic effects in offspring after alterations to maternal or early post‐natal diets. Evidence in humans is largely limited to observational and quasi‐experimental situations such as maternal famine exposure [19].

Early exposure to detrimental factors may increase the risk of chronic diseases later in life. This theory is known as the developmental origin of health and disease (DOHaD) [20]. However, post‐natal exposure to environmental factors may also contribute to obesity. A study by Mitchell et al. [21] analyzed a group of prenatal and current factors and exposures influencing BMI in children and adolescents in a large international sample of 65,721 children (27 centers, 15 countries) and 189,282 adolescents (70 centers, 35 countries). In children and adolescents, factors associated with BMI included birth weight and variables related to food intake and physical activity. In children, early life exposures (e.g., antibiotics, paracetamol, and breastfeeding) were also associated with BMI. Findings of this study suggested that current behaviors had larger effects on variation in BMI than early exposures.

The exposure to a large number of environmental factors that modulate epigenetic mechanisms has been associated with childhood obesity, including nutrients, diverse chemical substances, and pollutants. Endocrine disruptors (EDs) are defined by the Endocrine Society as “exogenous chemicals, or mixture of chemicals, that interfere with any aspect of hormone action” [22]. These substances have structural similarity to hormones, and their mechanisms include altering normal hormone concentrations, inhibiting or stimulating the production and metabolism of hormones, or changing hormones’ movement through the body [23]. These substances are classified as nonpersistent pollutants, such as bisphenol A (BPA) and phthalates, which may be present in food and water, and persistent pollutants that include tributyltin, dichlorodiphenyltrichloroethane, parabens, polycyclic aromatic hydrocarbon mixture, and inorganic arsenic [21, 23].

The present study aims to assess the evidence on the role of epigenetic mechanisms in obesity in children and adolescents, and the possible contribution of environmental factors, including ED compounds, to this association [24, 26].

## 2 Methods

This systematic review was completed according to the Preferred Reporting Items for Systematic Reviews and Meta‐Analyses (PRISMA) statement.

### 2.1 Literature Search Strategy

We conducted a literature search using public databases (PubMed and Scopus). Eligibility criteria included: (1) original observational studies; (2) original experimental human studies; and (3) meta‐analyses addressing the association between epigenetic mechanisms and obesity and related anthropometric traits. Epigenetic mechanisms included analysis of DNA methylation, histone modifications, and/or nc RNAs using appropriate techniques in tissues and/or fluids of children and adolescents 0–18 years of age. Studies had to use untargeted analytical methods followed by validation and/or replication.

For the search strategy, the following terms and their combinations were used: “obesity,” “BMI,” “body composition,” “overweight,” “adipose tissue,” “adiposity,” “childhood,” “endocrine disruptors,” “children,” “childhood obesity,” “teen obesity,” “adolescence,” “epigenetic,” “DNA methylation,” “histone modification,” “epigenetic marks,” “diet,” “bisphenol A,” “phthalates,” “nutritional status.” Studies had to be published in English in peer‐reviewed journals between 2013 and 2023. The last search was conducted on August 20th, 2024.

Duplicates and studies in languages other than English were removed. References were assessed independently by at least two reviewers. Discordant opinions were discussed with a third reviewer. Data extraction was conducted according to the PECO (population, exposure, comparison, and outcome) approach. We collected population characteristics (age group, country of origin or ancestry, sample size), epigenetic mechanism (DNA methylation, histone modification, or nc RNA analysis), analytic technique, main outcome of the study, and validation.

## 3 Results

**FIGURE 1:**
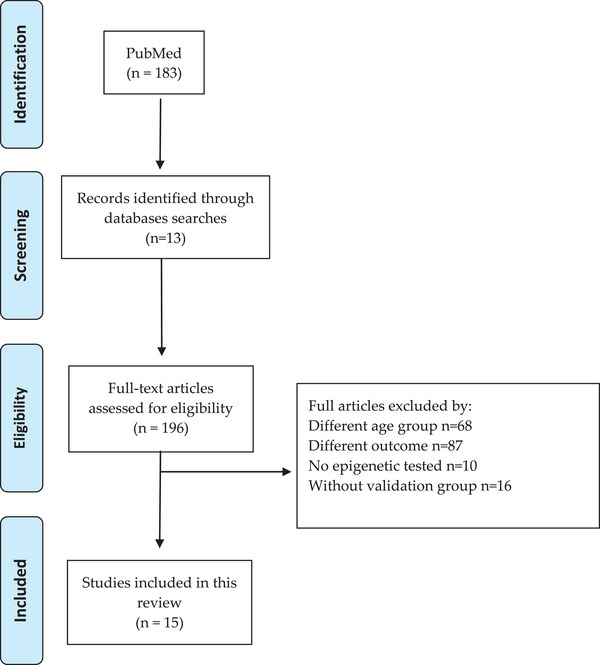
PRISMA 2020 flow chart describing the screening process.

The search retrieved a total of 183 articles for screening. Figure 1 shows the selection process of eligible references, resulting in 15 publications that fulfilled the inclusion criteria. Among the studies meeting the inclusion criteria, 54% were conducted in European populations (eight studies), 20% in Asian populations (three studies), and 6.5% each in North American (one study), Oceanian (one study), Hispanic, Indigenous, and European mixed populations (one study), and Asian, Turkish, and European mixed populations (one study).

Table 1 shows two meta‐analyses and systematic reviews on the association between epigenetic mechanisms and obesity in children [18, 28]. Table 2 shows seven studies that analyzed the association between DNA methylation patterns and obesity in children, using an untargeted approach and validation [29, 30, 31, 32, 33, 34, 35]. Table 3 contains six studies analyzing the association between RNAs (miRNAs and lncRNAs) and obesity, including a validation phase associated with obesity [16, 36, 37, 38, 39, 40]. One of these studies is a systematic review that included seven studies on the association between miRNA and obesity in children using different analytical methods. In addition, three original studies not included in the systematic review were eligible for this review.

**TABLE 1:** Meta‐analyses and systematic reviews on the association between epigenetic mechanisms and childhood obesity.

Author, year	Epigenetic mechanisms	Age	Sample size	Population	Ancestry	Analytical method, tissue	Phenotype	Outcome
Alfano et al., 2023 [18]	DNA methylation	1 year	2003 from six studies	Children	European	Illumina Human Methylation 450K/EPIC‐array. Associations of cord blood DNA methylation.	Rapid growth at 1 year	Forty‐seven CpGs were associated with rapid weight growth at suggested *p*‐value (*p*‐value<1x10^−05^), and three passed the genome‐wide significance (*p*‐value<1.25x10^−07^)
Vehmeijer et al., 2020 [28]	DNA methylation	2−18 years	4133 from 23 studies	Children and adolescents	European, African, Hispanic, and Indigenous people	Illumina Infinium HumanMethylation450 BeadChip assay. Epigenome‐wide, cord blood and whole blood.	BMI	DNA methylation at three CpGs, each in a different age range, was associated with BMI (*p*<1.06×10^−7^). DNA methylation at nine additional CpGs in the cross‐sectional childhood model was associated with BMI.

Abbreviations: BMI, body mass index; CpG, citosine followed by a guanine nucleotide and bound by a phosphate group.

**TABLE 2:** Epigenome‐wide studies on the association between DNA methylation and obesity‐related traits in children and adolescents.

Author, year	Epigenetic mechanism	Age	Sample size (*n*)	Participants	Country, ancestry	Analytical method, tissue or fluid	Phenotype	Outcome
Gagné‐Ouellet et al., 2020 [29]	DNA methylation	3 years	473 participants: 286 discovery cohort 187 validation cohort	Mother−children dyads	US European	Illumina HumanMethylation 450K/EPIC. Gestational age at delivery, sex of the baby, and age at follow‐up were included in the regression models as covariates. Analyses were also adjusted for cell composition Heterogeneity. Full‐term placenta.	BMI, skinfolds	BMI and skinfolds sum at 3 years were statistically associated with lower methylation at cg22436429 and cg22593959 in placenta. Genomic regions at the *BMPR1B*, *SKAP2*, *MAGI2*, and *FMN1* genes were associated with BMI at 3 years of age, and skinfold thickness.
Kaufman et al., 2018 [30]	DNA methylation	Discovery cohort 11.5±1.9 years Replication cohort 10.7±2.0 years	234 participants: 160 discovery cohort 74 replication cohort	Children	US	Illumina Infinium HumanMethylation450 BeadChip assay. Epigenome‐wide, Adjustment for age, sex, race, and cell type. Saliva.	BMI	DNA methylations at six CpGs were statistically associated with BMI. Eight of the methylation sites were in genes previously associated with obesity risk.
van Dijk et al., 2018 [31]	DNA methylation	5 years	442 participants	Children	Australian	Epigenome‐wide DNA methylation (Illumina array, Infinitum Human Methylation 450 bead chips). Neonatal spot blots. Adjustment for sex and age.	SD BMI scores, skinfold thickness, body fat, waist circumference, and hip‐waist ratio	No individual methylation sites at birth were associated with obesity at 5 years of age. DNA methylation in 69 genomic regions at birth was associated with BMI z‐scores at 5 years of age. Methylation changes were generally small (<5%). DNA methylation was associated with maternal smoking and birth weight.
Samblas et al., 2018 [32]	DNA methylation	10.7±0.4 years cases 10.8±0.3 years controls	24 participants: 12 children with obesity 12 children without obesity	Children	Spain	Epigenome‐wide DNA methylation (Illumina array, Infinitum Human Methylation 450 bead chips). Peripheral blood. Adjusted for age and sex.	Obesity defined as BMI>97th percentile of Spain's reference	Genome‐wide analysis identified 734 CpGs (783 genes) differentially methylated between cases and controls. None reached epigenome‐wide significance.
Lillycrop et al., 2017 [3^3^]	DNA methylation	Newborns and 6 years	21 participants	Children	UK	Epigenome‐wide DNA methylation (chromatin immunoprecipitation, DNA microarray, Agilent Human promoter Whole‐Genome ChIP‐on‐chip array). Umbilical cord samples.	Fat mass measured by DEXA	An association was found between the level of CpG methylation at birth within the promoter of the long noncoding RNA *ANRIL* (encoded at *CDKN2A*) and childhood adiposity at age 6 years. This finding was consistent with other three studies. Perinatal methylation at loci relevant to gene function may be a marker of future adiposity in children.
Eriksson et al., 2015 [34]	DNA methylation	9−13 years	69 participants: 24 girls with obesity, 23 preadolescent girls without obesity as well as 11 boys with obesity and 11 preadolescent boys without obesity	Children	Greece	Epigenome‐wide DNA methylation. Blood cells.	Obesity and overweight	No difference was found in the methylation patterns between study groups.
Ding et al., 2015 [35]	DNA methylation	3−6 years	32 participants	Preschool children	China	Epigenome‐wide DNA methylation, methylated DNA immunoprecipitation sequencing, NimbleGen MeDIP chip.	Obesity	Compared with children without obesity, children with obesity had 251 promoters and 575 CGIs demethylated, and 141 promoters and 277,251 promoters hypermethylated. Four genes (*FZD7*,* PRLHR, EXOSC4*, and *EIF6*) with differential promoter methylation were validated.

Abbreviations: BMI, body mass index; CGI, CpG island; CpG, cytosine nucleotide followed by a guanine; DEXA, dual energy X‐ray absorptiometry.

**TABLE 3:** Studies on the association between noncoding RNAs and childhood obesity.

Author, year	Sample size (*n*)	Age	Population, country	microRNA analysis method, tissue	Phenotype	Findings
Rico‐Flórez et al., 2020 [36]	687 participants from seven studies. Systematic review	2−16 years	European, Chinese, and Turkish	Plasma, serum, whole blood.	Obesity	A heterogeneous group microRNAs was associated with obesity in children. miR‐15b‐5p, miR‐486‐5p, and hsa‐miR‐122‐5p were associated with obesity.
Mas‐Parés et al., 2019 [37]	10 children: 5 SGA‐CU and 5 SGA‐nonCU Validation: 64 children	0−6 years	Spain	752 microRNAs (qPCR, miRCURY LNAuniversal RT microRNA PCR human panels I and II Exiqon). Cord blood.	Catch‐up growth, weight, and waist and hip circumference	Twelve microRNAs were differentially expressed between groups of children (miR‐128‐3p, miR‐222‐5p, miR‐300, miR‐374b‐3p, miR‐501‐3p, miR‐548c‐5p, miR‐576‐5p, miR‐628‐5p, miR‐770‐5p, miR‐873‐5p, miR‐876‐3p, and miR‐940) (all nominal *p*<0.05). Among these 12 microRNAs, miR‐576‐5p showed significant positive associations with weight and catch‐up weight at 12 months and weight and waist circumference at 6 years.
Iacomino et al., 2019 [38]	308 children: 159 with obesity and 149 controls Validation: 189 participants	12 years	Belgium, Cyprus, Estonia, Germany, Hungary, Italy, Spain, and Sweden	372 microRNAs. Peripheral Blood.	Obesity and BMI	Nine microRNAs were differentially expressed between children with obesity and controls (*p*<0.05) and five (miR‐10b‐5p, miR‐215‐5p, miR‐501‐5p, miR‐551a, and miR‐874‐3p) correlated with BMI. Validation: miR‐10b‐5p, miR‐191‐3p, miR‐215‐5p, miR‐501‐5p, miR‐551a, miR‐874‐3p quantified by RT‐qPCR were differentially expressed in children with and without obesity.
Marzano et al., 2018 [39]	16 children: 8 with obesity and 8 without obesity	11.9 years	Italy	Genome‐wide microRNA expression profile (array, Illumina microRNA sequencing platform). Sex‐ and age‐adjusted BMI>95th percentile. Peripheral blood.	Obesity	MicroRNAs (miR‐92a‐3p, miR‐122‐5p, miR‐423‐5p, miR‐484, miR‐486‐3p and miR‐532, miR‐181b‐5p) were differentially expressed in children with obesity compared to children without obesity (FDR *p*<0.05).
Liu et al., 2018 [16]	8 children 4 with obesity 4 without obesity	8 years	China	Genome‐wide long noncoding RNA expression profile (Kan Chen Biotech). Adipose tissue.	Obesity, waist circumference, and waist‐hip ratio	1268 long noncoding RNAs had >2‐fold differences between children with and without obesity. Pathway analysis identified metabolic processes related to obesity. Validation showed correlation between some lncRNAs and BMI in 64 children lncRNARP11‐20G13.3.
Cui et al., 2018 [40]	18 children: 9 with obesity and 9 without obesity	5 years	China	Genome‐wide microRNA expression profile (array, Illumina microRNA sequencing platform). Peripheral blood.	Obesity defined as: WHO BMI SD>2 (for age <60 months) and WHO BMI SD>3 (for age>60 months)	Ninety‐four microRNAs >2‐fold change in expression between children with obesity and controls (*p*<0.05). Validation: miR‐222, miR‐486, miR‐146b, miR‐146a, miR‐20a, miR‐15b and miR‐26b, miR‐197 (RT‐qPCR) were differentially expressed in 100 children with obesity and 146 controls (*p*<0.05) and significantly correlated with BMI (*p*<0.05).

Abbreviations: BMI, body mass index; RT‐PCR, reverse transcription polymerase chain reaction; SGA‐CU, small for gestational age with catch‐up; SGA‐nonCU, small for gestational age non ‐catch‐up.

No studies on histone modifications, including a validation phase, were found.

Regarding the meta‐analyses and systematic reviews on the association between epigenetic mechanisms and obesity (Table 1), the study by Alfano et al. [18] included six European‐based cohorts and analyzed the association between DNA methylation in cord blood and rapid growth at 1 year of age.

The study by Vehmeijer et al. [28] included 23 studies and investigated the association between DNA methylation in cord blood or in circulating cells, and obesity‐related traits, using a DNA methylation microarray that includes > 450,000 CpG (Illumina HumanMethylation 450K/EPIC‐array). This meta‐analysis included children from 2 to 18 years of age.

Studies on DNA methylation used an untargeted approach (epigenome‐wide analysis) followed by validation and/or replication. The most analyzed substrates were blood cells or derived fluids (plasma or serum), saliva, and placental tissue. Six studies analyzed RNAs; of these, five measured nc RNAs in blood cells or fluids, and one analyzed them in adipose tissue. Most of the studies were conducted among participants of European ancestry. No studies were found on the effect or association of ED compounds or other environmental factors on epigenetic mechanisms associated with childhood obesity.

### 3.1 DNA Methylation in Childhood Obesity

The most analyzed mechanism associated with childhood obesity and related traits was DNA methylation. DNA methylation was analyzed mainly in blood samples, although placental tissue has been used for assessing the association of methylation patterns and children's characteristics [29]. The understanding of this mark in children is important, given that methylation patterns are persistent and established in early life.

We identified two meta‐analyses addressing the association between DNA methylation and phenotypes related to obesity in children and/or adolescents (Table 1). The study by Vehmeijer et al. [28] found a significant association between DNA methylation at three CpGs and BMI. In addition, this study showed an increase in BMI associated with an increase in methylation, respectively. The study by Alfano et al. [18] found a suggestive association between DNA methylation in 47 CpGs analyzed in cord blood cells and weight gain at 1 year of age in six studies.

DNA methylation has been studied using different technologies that allow for the analysis of (1) small groups of methylation sites within selected regions regulating the expression of a candidate gene; (2) a group of regulatory sites associated with genes that have been related to obesity; and (3) more recently, using tools for epigenome‐wide analysis that includes thousands of CpG regions. One of the most commonly used arrays is Illumina Infinium Human Methylation [16, 19, 41]. This approach, like other omics techniques, has the potential for the discovery of novel regulatory regions and their associated genes.

The seven eligible studies on epigenome‐wide studies on the association between DNA methylation and obesity‐related traits (Table 2) were conducted in two phases: (1) the discovery of differentially methylated regions using an epigenome‐wide approach; and (2) replication in a similar cohort, or validation of findings, using a small‐scale method or a technique that may explore the underlying mechanism explaining the association. The replication and/or validation are strongly recommended to reduce the probability of false‐positive results. The selected studies show heterogeneity in the research question, although all of them analyzed the relationship between DNA methylation and obesity and related traits using an epigenome approach. Findings suggest associations between DNA methylation in different tissues and adiposity at different ages in children and adolescents, although the identified sites differ across studies.

Three studies found an association between methylation in blood DNA at birth, using cord blood and blood from neonatal spot blots, and obesity‐associated phenotypes (BMI, fat mass) in later childhood [29, 31, 33] (Table 2). The other four studies investigated the association between DNA methylation in saliva or blood and obesity in children, reporting significant associations with diverse regions of the genome, including regulatory regions of genes associated with obesity.

### 3.2 MicroRNA and Other Noncoding RNA

A systematic review (684 children) on miRNAs associated with childhood obesity analyzed miRNAs present in blood or blood fractions using different detection methods, mainly RT‐PCR [36] (Table 3). There was large heterogeneity in the methods used for the study of miRNAs, which prevents comparing studies testing different sets of miRNAs across different cells or fluids. This systematic review concluded that miR‐15b‐5p, miR‐486‐5p, and hsa‐miR‐122‐5p in blood have a consistent association with childhood obesity. Studies that used blood cell or blood‐derived fluids are cross‐sectional, used different detection methods (different arrays), and included children of different ages. Thus, although all the studies identified differentially expressed miRNAs between children and adolescents with obesity, the set of miRNAs varied across studies.

A study analyzing lncRNAs in adipose tissue of children with and without obesity [16] found 1268 differentially expressed transcripts with >2‐fold change in expression between children with obesity and controls (*p*<0.05). Results suggest that 10 lncRNAs are involved in biological processes relevant for obesity, including immune response, inflammatory response, fatty acid biosynthetic process, low‐density lipoprotein particle remodeling, and AMPK signaling. A group of lncRNAs were validated in a different sample of 64 children using a different detection method. The bioinformatics analysis of these data showed that these transcripts were involved in pathways associated with obesity and inflammation.

## 4 Discussion

The study of epigenetic mechanisms is challenging due to factors such as the tissue‐specific nature of dynamic processes, the difficulty of assessing the exposure to a large number of environmental factors that regulate epigenetic mechanisms, and the changes that occur throughout life. In this review, which aimed to assess the role of epigenetic mechanisms in obesity in children and adolescents, we identified 15 eligible studies.

Two meta‐analyses on the association between DNA methylation and obesity‐related phenotypes analyzed different traits. The study by Alfano et al. [18] investigated the association between rapid weight gain and DNA methylation. The study by Vehmeijer et al. [28] analyzed the association with BMI. In the first study, three CpGs (cg14459032, cg25953130 annotated to *ARID5B*, and cg00049440 annotated to the transcription factor *KLF9*) showed association. *ARID5B* is a gene that encodes for an enzyme involved in histone modifications, and *KLF9* encodes for a transcription factor. None of these genes or related CpGs have been associated with obesity. According to these findings, there is an association between BMI and related traits in children. However, the available studies differ significantly in study design, age group, data analysis, and other characteristics of the studies, which makes it difficult to draw conclusions. These studies investigated the association between DNA methylation at specific CpG, although no information on factors regulating this mechanism, such as diet or other environmental factors, is considered.

Studies using a targeted approach have tested DNA methylation in *a priori* selected regions regulating obesity‐related genes. Findings of these studies showed differences in DNA methylation marks associated with childhood obesity in leptin (*LEP*), leptin receptor (*LEPR*) [42, 43] adiponectin (*ADIPOQ*), adiponectin receptors (*ADIPOQR*), peroxisome proliferator‐activated receptor gamma (*PPARG*) hypoxia‐inducible factor 3α (*HIF3A*), and long interspersed nuclear element‐1 (*L1RE1*). The methylation patterns at the promoter regions of these genes that are relevant for adipose tissue biology, appetite regulation, and insulin sensitivity differ between children with and without obesity. Although the findings of these studies show a biological mechanism that associates DNA methylation with BMI and obesity, these CpGs have not been identified in studies studying large samples using epigenome‐wide approach.

The role of environmental factors influencing epigenetic mechanisms requires further research. To date, few studies have investigated these effects on human obesity. The study by Geraghty et al. [44] tested the effect of a dietary intervention with different glycemic indices during pregnancy and analyzed DNA methylation in saliva. According to these results, there was no effect with this dietary intervention during pregnancy, and no association between maternal age, weight, or BMI during pregnancy and offspring DNA methylation was found (*p* > 0.01). No associations with child weight or adiposity at 5 years of age were identified; however, the change in body weight in children at 6 months was associated with the methylome. The study by Lv et al. [45] investigated the association between exposure to endocrine‐disrupting chemicals (EDCs) during pregnancy, BMIz in children, and DNA methylation. Urinary concentrations of seven EDCs were positively associated with BMIz in the first trimester. DNA methylation was measured using an epigenome‐wide array. This study found a total of 641 differential DNA methylation positions significantly associated with elevated BMIz. Twelve CpG sites annotated to the genes *DUXA*, *TMEM132C*, *SEC13*, *ID4*, *GRM4*, *C2CD2*, *PRAC1 & PRAC2*, *TSPAN6*, and *DNAH10* mediated the associations between urine EDCs and elevated BMIz (*p* < 0.05). These findings suggest that exposure to EDCs during pregnancy may be related to childhood obesity, and DNA methylation may be a mediator mechanism. The study by Khodasevich et al. [46] investigated the association between prenatal exposure to BPA, triclosan, benzophenone‐3, methyl‐paraben, propyl‐paraben, and butyl‐paraben, as well as 11 phthalate metabolites and DNA methylation in cord blood, and circulating cells in children at 9 and 14 years of age. This study identified CpGs associated with the prenatal levels of the mentioned pollutants in a sex‐specific manner. This study did not address any obesity‐related phenotype.

The studies on RNA transcripts have identified functional differences between tissues of participants with and without obesity. Thus, many of the miRNAs differentially expressed in visceral adipose tissue of children with obesity have been reported to be enriched in pathways related to lipid metabolism, such as fatty acid oxidation, ketogenesis, lipogenesis, and lipid uptake, which could be directly related to increased adipogenesis, fat mass gain, and liver steatosis [47, 48]. The study by Liu et al. [16] compared gene expression in adipose tissue of children and adolescents with and without obesity using differentially expressed genes from two publicly available datasets, GSE9624 and GSE88837. This study analyzed the potential miRNAs that regulate the transcripts. The identified genes are regulated by miR‐16‐5p, miR‐124‐3p, miR‐103a‐3p, and miR‐107. The latter is the miRNA with the maximum number of miRNA–mRNA interaction pairs. The identified miRNAs were significantly enriched in pathways such as lipid metabolism, immune response, vascular inflammation, and brain development, and were associated with prediabetes, diabetic nephropathy, depression, solid tumors, and multiple sclerosis. A study by Rico‐Flórez et al. [49] found that miRNA hsa‐miR‐122‐5p was overexpressed in a group of children with obesity. This study suggests that the amount of adipose tissue may be associated with hsa‐miR‐15b‐5p, hsa‐miR‐222‐3p, hsa‐miR‐122‐5p, and hsamiR‐191–5p in blood.

Wu and Perng [8] described three relevant moments of exposure associated with childhood obesity: (1) in‐utero, including germinal, embryonic, and fetal periods; (2) infancy, ranging from birth to 2 years of age; and (3) early childhood, ranging from 2 to 6 years of age. The current evidence on epigenetic mechanisms associated with childhood obesity addressed a wide variety of questions using different study designs and technological resources. An important consideration is that most studies have been conducted in readily available tissues and fluids, such as blood cells, plasma, and serum, and, in very few cases, adipose tissue, which may not resemble the epigenetic marks and gene activity in other tissues. In addition, studies conducted in young children require less invasive methods. Epigenetic studies that use blood samples are advantageous because they are accessible and allow for minimally invasive collection, making them suitable for large‐scale studies and research involving children. However, blood samples may not fully reflect tissue‐specific modifications. Evaluating adipose tissue offers more appropriate insights into the epigenetic regulation of metabolic pathways related to childhood obesity, although this approach can be a challenge due to its invasiveness, cell‐type heterogeneity, and limited applicability in specific populations [50]. These factors highlight the importance of selecting the appropriate sample types for research purposes.

The available studies on DNA methylation suggest that patterns found in selected genes and genomic regions are associated with obesity‐related phenotypes in children. Some of these studies have found that DNA methylation patterns in cord blood and placental tissue are associated with growth, and adiposity markers in later childhood [5, 19]. While these studies are longitudinal, it is difficult to establish a causal relationship between epigenetic marks and phenotypes. Information on the role of histone modifications in childhood obesity requires further research, since very limited information is available from humans.

The role of nc RNAs in childhood obesity requires further research to integrate results obtained from different platforms and study designs. These groups of molecules are promising as potential biomarkers for adult diseases and may be of interest for childhood obesity. Lastly, research on the role of the different environmental factors in childhood obesity is required. Animal models have shown the mechanisms exerted by abundant substances such as pollutants, and human research is necessary to define their role in obesity and related diseases. Current evidence of the effects of EDCs or other compounds on epigenetic mechanisms linked to obesity come from animal models. Observational studies in humans have identified an association between these compounds and adiposity, although no epigenetic mechanisms have been tested in children or adolescents. There is increasing evidence that exposure to some environmental compounds during fetal development has long‐term programming effects. EDCs that methylate DNA can target the cytosine residues located in CpG dinucleotides by adding a methyl group [41]. Timing and dose of exposure may have different effects.

Studies conducted in animal models identified the transgenerational effects of substances such as BPA, organochlorine compounds, phthalates, polybrominated biphenyl, polycyclic aromatic hydrocarbons, parabens, benzoic acid, and polyfluoroalkyl. These substances are considered as obesogenic, as they may produce abnormal adipocytes and reduce insulin sensitivity and thermogenic capacity [24, 25]. They may also induce abnormalities in lipid metabolism and unhealthy adipogenesis with reduced insulin sensitivity and decreased thermogenic capacity [22]. These effects may be explained by the biological activity of the retinoid X receptor‐alpha/peroxisome‐proliferator activated receptor gamma and the glucocorticoid receptor.

A systematic review and meta‐analysis on the association between a group of EDCs (BPA) and obesity in children and adults identified 16 studies that investigated the association between BPA exposure and obesity in children and adolescents. Findings across studies were inconsistent. Subanalyses by sex and age showed statistically significant associations in girls [22]. Kim et al. [51] investigated the association between BPA exposure and childhood obesity. The results showed that the group with a relatively high exposure had a statistically significant higher risk of childhood obesity than the group with a relatively lower exposure. A similar finding was reported by Wang et al. [52] in Chinese girls between 8 and 11 years. Overall, studies suggest that the association between BPA and BMI in children was age‐dependent and gender‐dependent. The best‐characterized compound with transgenerational effects in humans is tobacco smoke. Maternal smoking during pregnancy is associated with lower birth weight and increased risk of obesity in children [19]. The possible role of epigenetic mechanisms in these effects remains to be elucidated. Despite increasing interest in the role of EDCs in the etiology of childhood obesity, there remains a significant gap in the literature addressing their potential impact through epigenetic mechanisms. Limited evidence exists on how prenatal and early postnatal exposure to EDCs may alter the epigenetic landscape in ways that predispose children to metabolic dysregulation and increased adiposity. Future research should include longitudinal cohort designs with well‐characterized exposure evaluations, focus tissue analyses when possible, and omics sciences such as epigenomic, transcriptomic, and metabolomic data profiles. These approaches are necessary to explain the complex relationship between environmental exposures and gene regulation during critical developmental frames.

Variability in the design of epigenetic studies related to childhood obesity has significant challenges for cross‐study comparisons and meta‐analyses. Differences in detection methods (such as the use of miRNA microarrays, methylation arrays, or next‐generation sequencing), lack of validation protocols, diversity in the samples included (blood or tissue), and predominantly Caucasian study populations, limit the reproducibility and generalizability of findings. Although these limitations are often recognized, their data integration and interpretation implications remain unexplored. However, advances in cutting‐edge technology and bioinformatics tools require standardized protocols, provide new opportunities to reduce confounding factors, and enable large‐scale, multicohort analyses with greater precision and relevance [53].

### 4.1 Strengths and Limitations

The main strength of this study is the inclusion of epigenome‐wide studies with validation on the association between epigenetics. The use of methods that analyze methylation across the whole genome, followed by a validation phase, allows the identification of novel regions associated with the phenotypes of interest.

Regarding limitations, the heterogeneity of study designs and detection methods in the identified references limits comparability and aggregated analyses. Included studies had relatively small sample sizes, and most lacked information on exposures that may be relevant for the study of childhood obesity. In addition, most of the studies were conducted only in Caucasian children, and very limited information is available on other populations. The latter is relevant not only because of genetic diversity across groups, but also for potential variability of environmental factors. As shown in this review, there is active research on the association between childhood obesity and epigenetic mechanisms, and on the association between exposure to different pollutants and childhood obesity; however, these elements have not been integrated in studies with humans.

Lack of diversity among the studied populations represents a significant limitation when attempting to generalize findings related to epigenetic mechanisms and childhood obesity. Most available data are from European cohorts, with minimal representation of Hispanic, Indigenous, African, or mixed‐ancestry populations. Due to the already‐known genetic, environmental, and sociocultural differences across populations, increasing research efforts to include underrepresented groups is indispensable. These considerations could improve the precision and applicability of conclusions, ensuring future therapeutic strategies for different populations.

## 5 Conclusions

There is an imperative need to develop research into the epigenetic basis of childhood obesity. Epigenetic mechanisms are regulated by a large variety of environmental factors, particularly at early stages of life. DNA methylation is the most analyzed epigenetic mechanism associated with childhood obesity. Despite the heterogeneity of study designs and analytical methods, there appears to be an association between DNA methylation in blood cells, adipose tissue and placenta, and childhood obesity. Circulating miRNAs in blood and other tissues also present an association with childhood obesity. The biological activity of these molecules in obesity requires further research. The contribution of histone modifications to childhood obesity has been underexplored compared to DNA methylation and miRNAs. The role of persistent and nonpersistent pollutants, as well as EDCs, on the regulation of epigenetic mechanisms involved in childhood obesity remains to be investigated. Future research should consider the advantages and limitations of current detection or analysis methods, biological sample types, population diversity, as well as longitudinal studies, with a proper sample size. Furthermore, new research should be methodologically consistent and include cross‐cohort validation of results and the development of reproducible and scalable methodologies to ensure robust and clinically meaningful results.

## Author Contributions

G.L.‐R., G.K.G.‐Q., and F.J.L.‐A. conducted the search, extracted data, and contributed to writing the manuscript. M.E.T. conceptualized the entire study and wrote the manuscript.

## Conflicts of Interest

The authors declare no conflicts of interest.

## Data Availability

Data sharing is not applicable to this article as no datasets were generated or analyzed during the current study.
